# Predictive testing for Huntington’s disease in a digital age; patient power with potential pitfalls

**DOI:** 10.1038/s41431-025-01793-1

**Published:** 2025-01-29

**Authors:** V. Mocanu, SG Lindquist, LE Hjermind, JL Heilmann, R. MacLeod, N. Lahiri

**Affiliations:** 1 City St. George’s University, School of Health & Medical Sciences, London, UK; 2 Department of Clinical Genetics, Rigshospitalet, Copenhagen University Hospital, Copenhagen, Denmark; 3 Danish Dementia Research Centre, Department of Neurology, Rigshospitalet, Copenhagen University Hospital, Copenhagen, Denmark; 4 Department of Clinical Medicine, University of Copenhagen, Copenhagen, Denmark; 5 Huntington’s Disease Youth Organization, Livonia, MI USA; 6 Manchester Centre for Genomic Medicine, Manchester, UK; 7 Division of Evolution, Infection and Genomics, School of Biological Sciences, Faculty of Biology, Medicine and Health, Manchester Academic Health Science Centre, University of Manchester, Manchester, UK; 8 SW Thames Centre for Genomic Medicine, St. George’s University Hospital, London, UK

**Keywords:** Health policy, Ethics

## Abstract

Advances in digital healthcare impacts genetic counselling and testing for hereditary disorders, including Huntington’s Disease. The Danish Digital Health Strategy was implemented from 2018 but has recently announced an extension to include the automatic release of all results, including genomic laboratory reports. The European Huntington’s Disease Network (EHDN) Working Group for Genetic Counselling and Testing have reviewed the existing Recommendations for Predictive Testing in Huntington’s Disease and the literature concerning digital health records and make recommendations to maintain the integrity of genetic counselling practise.

## Introduction

The Danish Digital Health Strategy, implemented from 2018, is an example of advanced and integrated digitization allowing patients immediate digital access to most data (https://sundhedsdatastyrelsen.dk/da/english/digital_health_solutions/digital_health_strategy). In February 2025 a new database will be established enabling automatic release of results from all pathology including genetic tests.

The European Huntington’s Disease Network and European Huntington’s Association responded to concerns raised and here we outline the potential implications for Huntington’s Disease.

Huntington’s disease (HD) is a dominantly inherited neurodegenerative condition. Due to the inherited nature, an absence of preventative measures or disease modifying therapies and high variability in phenotype and age of onset; predictive testing for at risk family members follows international best practise recommendations [1]. These recommendations establish minimum standards for genetic counselling, to prepare at risk individuals and provide a reference point for ethical and clinical dilemmas. The aim is to ensure that people at risk of HD are well informed and supported at all stages of their decision-making process; before, during and after testing and that the process is robust to minimise post-test regret and the risk of severe adverse reactions.

They serve as a model for other inherited late-onset neurological disorders.

Digitization of health records allows prompt access to health information but raises several concerns in relation to predictive testing. Unregulated access to results could lead to misunderstanding by patients and healthcare professionals; there are risks that pre-symptomatic individuals are mis-labelled as affected, and there is potential for discrimination through inappropriate sharing of a predictive test result with prospective employers or insurance companies as part of a wider health care record release. Whilst these concerns are not specific to Denmark, their strategy to include genetic results directly impacts predictive test recommendations (Table 1). It is essential to avoid miscommunication of this life-altering information and provide timely psychological support. In addition, an individual has the right to postpone or cancel disclosure and the strategy reduces patient autonomy, potentially coercing individuals to receive a result that they are not ready for (Table 2).

**Table 1:** Summary of recommendations related to results from current international recommendations for predictive testing in HD.

Theme	Recommendation
Access to the test	• REC2 The decision to take the test is the sole choice of the person concerned. No requests from third parties, be they family or otherwise, should be considered. • REC2.3 Persons should not be discriminated against in any way as a result of genetic testing for HD. • REC2.4 Extreme care should be exercised when testing would provide information about another person who has not requested the test. • REC2.6 Testing for HD should not form part of a routine blood investigation without specific permission. • REC2.7 Ownership of test results must remain with the person who requested the test.
Support during testing process	• REC3.1 The counselling unit should plan with the participant a follow up protocol which provides support during the pre- and post- test stages.
Recommendation on communication of information	• REC4.1 The laboratory performing the results should not communicate the results to the counselling team until very close to the time results are given to the participant. • REC4.2 As a rule, members of the counselling team or technical staff should not communicate any information concerning the test and its results to third parties without the explicit permission of the person tested. • REC4.4 Care should be taken regarding access to clinical reports of the test results.
General information	• REC5.2.6 The predictive test indicates whether someone has or has not inherited the gene mutation, but it does not make a clinical diagnosis of HD if the gene expansion is present. • REC5.3.5 Potential socioeconomic consequences, including employment, legal care of and access to children, adoption eligibility, social security, data security and other problems which may occur because of disclosing the test result or family history.
The test and delivery of results	• REC8.2 The results of the test should be delivered as soon as reasonably possible after completion of the test, on a date agreed upon in advance between the center, the counsellor, and the person. • REC8.3 The manner in which results will be delivered should be discussed between the counselling team and person. • REC8.4 The participant has the right to decide at any point that the result should not be given to him/her. • REC8.5 The results of the test should be given personally by the counsellor to the person and his/her companion. In geographically remote areas the results session may be arranged by prior agreement with a clinician known locally to the participant. No results should ever be given by telephone or by mail. The counsellor must have sufficient time to discuss any questions with the person. • REC 8.6 All post-test provisions must be available from the time the test results are given

Adapted from [1].

**Table 2:** Summary of concerns related to the potential negative impact of automatic results for predictive test result.

What could the impact of automatic release in relation to HD patients and families be?
- Coercion to receive the result.
- Lack of immediate support.
- Potential for acute distress, impulsivity, and suicidal behaviour
- Misunderstanding of the results (i.e. intermediate and reduced penetrance alleles).
- Potential for breaches of patient confidentiality and inadvertent data sharing.
- Those who do not carry the HD gene expansion may be less likely to attend a follow up appointment and therefore miss out on support

Studies following predictive testing demonstrate hopelessness [2] and adverse effects on mental health can occur [3]. This is also true of gene-negative results where post-test counselling remains beneficial [4]. Whilst many people adjust to their result, a small but significant percentage experience serious adverse reactions including suicidality [5]. These people received their results in person according to current recommendations. The number of adverse events could increase in the absence of professional support at the point of accessing results.

## Automatic release of healthcare results

Several themes emerged from a literature review with the search terms ‘electronic health record AND patient online access results [6]. There are limited large-scale reports and the literature specific to genomics is sparse.

### Patient engagement

Online access to healthcare results makes it easier for patients to obtain information [7] increasing patient engagement resulting in them being better informed, better prepared for appointments, and more likely to ask pertinent questions. Using electronic health records leads to increased information sharing with patients, engagement with management plans and increased clarity of information [8].

### Potential severity of result

There are recommendations to limit digital access for more serious test results in the absence of appropriate follow-up [9] and apprehension from patients to receive potentially bad news results online without direct access to a clinician [10, 11].

### Timing of results

Some prefer to receive digital results as soon as possible in order to increase health transparency, to be reassured quickly or to begin processing bad news and prepare for a medical consultation [7]. In contrast, some do not want to learn more serious results online even if this results in a longer wait for results to be given in an appointment [9].

### Impact on wellbeing

There is general agreement that viewing results online is valuable, improves understanding and promotes patient autonomy [10] and there is demand for access to both raw data and reports [9]. However online access and automatic results release can increase anxiety and distress [12–15]. Results revealing a new diagnosis can lead to information seeking [12]. Interpretation of results is challenging and can result in health anxiety for insignificant results and increased clinician workload [13, 14]. There are also concerns about discovering unexpected information in online records [16].

### Potential for discrimination

There are concerns related to understanding of medical terminology and a potential for breaches of confidential health data [15]. Barriers to accessing digital platforms include poor health literacy, language and socioeconomic disparities thereby increasing health inequality [4, 17, 18]. People from certain minorities are less likely to be offered access to online portals by their clinician [19]. Women, English speakers, younger (25–39 years) people and those with private insurance are more likely to access [20].

The literature specific to genomics and digital records and automatic release of results is limited. It should be noted that genomic data is not always subject to access restrictions in digital records [17] and some HD speciality centres may take additional steps to prevent discrimination and protect confidentiality; omitting referral reasons, using pseudonyms, or retaining paper results [21]. This poses challenges due to increased workload, identification errors, inability to easily retrieve results and risk of missing records [22].

The recent CADRe framework highlights key components in consent for genetic testing and stresses the importance of a clear discussion of the timing and manner of return of results [18].

The MAGENTA study investigated the effects of omitting genetic counselling during testing for *BRCA1* and *BRCA2* with online release of results. There was no significant difference in distress in groups with no counselling but there were very few positive results which were spread across all groups [23].

There is disparity between medical professionals and the public regarding the utility and significance of results from direct-to-consumer genetic health testing companies but they are an existing example of automatic release of genomic results. They include disclaimers for consumers to acknowledge prior to release of results, recommend genetic counselling and consumers can exclude specific results, including *BRCA1 and BRCA2* and Alzheimer’s Disease. The engagement and understanding of these disclaimers is variable and there are examples of significant distress following receipt of results [24, 25].

## Discussion and recommendations

Digital advances are inevitable and to be supported but careful consideration of specific scenarios and flexibility in implementation is crucial.

Online access to patient data and results can increase patient engagement, adherence to management plans and assist in clarification of important information. However, benefits may not endure for all results and healthcare professionals and patients both advise against automatic release where the implication of the result could be serious, as is the case for HD predictive test results.

The potential severity, the timing of release of results and the potential for health inequalities have emerged as themes directly relevant to HD and we stress the importance of maintaining the integrity of existing recommendations for predictive testing.

The experiences from online testing in cancer genetics should be extrapolated with caution; they may allow access to screening programmes for early detection, early intervention and potentially disease modifying therapies where the results from predictive testing for adult onset neurodegenerative conditions do not and therefore adverse outcomes from receiving results may be greater, specifically to those already vulnerable [26].

We recommend clinician-controlled release of online results. Genetic test results are technical reports containing testing methods and validation and are intended for clinicians. Clinician controlled release maintains existing genetic counselling practise allowing a mutually convenient in person (or video) appointment to ensure communication of the result is clear and understood, that appropriate support and follow up are in place and to establish patient agreement to digital release.

This approach mitigates for those situations where patients choose not to receive their results.

Where a healthcare system lacks flexibility and mandates change; predictive testing protocols must be adapted to include discussion of and preparation for automatic results release.

Aspects to consider should include (Table 3); Patients should be familiarised with report templates and where to find relevant information. A clear timescale for results and how they will be notified (i.e. app notification, email, text message). Discussions could include avoidance of opening notifications inadvertently, though this may be challenging in practise. A notification could be ignored to wait for the results appointment, or the notification could open in a controlled manner with an agreed support person to digest the result prior to a carefully planned appointment. Contacts for clinical team the appropriate Huntington’s disease support organisation as well as local mental health crisis support would be essential.

**Table 3:** Physician controlled release of results is recommended.

Checklist for pre-test counselling where there is automatic release of predictive test results.
- When will the result be notified?
- When will the results appointment be?
- How will the result be notified?
- What will the report look like?
- Where is the relevant information pertaining to the result?
- Advise against inadvertent opening of the notification.
- Discuss options for dealing with an automatic result notification.
- Ensure contact details for clinician, HD advocacy group and mental health crisis support.

If automated release of genetic test results are mandated by a healthcare systems without flexibility then genetic counselling sessions could incorporate this checklist.

Appropriate information regarding access to the report by health professionals or agencies should also be given.

This approach, whilst aiming to maintain the integrity of genetic counselling does not mitigate all risk and increases the burden of careful co-ordination on patients, the laboratory, and clinical services.

## Conclusions

Online access to healthcare data has the potential to increase patient autonomy and engagement. However, an inflexible approach to automation paradoxically risks a patient centred approach and should be approached with caution. Implementation of healthcare digitisation should protect those seeking predictive testing for HD and other neurodegenerative conditions and maintain the integrity of genetic counselling recommendations. We recommend clinician-controlled release of genetic test results as opposed to automatic digital release and advise adequate consultation including with lay organisations. Research to ascertain the opinions of HD patients, their families and healthcare professionals regarding online access to genetic test results could be considered to shape future digitation strategies.
